# Effect of the Temperature-Emissivity Contrast on the Chemical Signal for Gas Plume Detection Using Thermal Image Data

**DOI:** 10.3390/s8106471

**Published:** 2008-10-21

**Authors:** Stephen Walsh, Larry Chilton, Mark Tardiff, Candace Metoyer

**Affiliations:** PO Box 999, Pacific Northwest National Laboratory, Richland, WA 99352 E-mail: (stephen.walsh@pnl.gov). E-mail: (lawrence.chilton@pnl.gov) E-mail: (mark.tardiff@pnl.gov). E-mail: (candace.metoyer@pnl.gov)

**Keywords:** plumes, signal, emissivity, clutter, temperature, LWIR, hyperspectral, Signal-to-Noise Ratio

## Abstract

Detecting and identifying weak gaseous plumes using thermal imaging data is complicated by many factors. These include variability due to atmosphere, ground and plume temperature, and background clutter. This paper presents an analysis of one formulation of the physics-based radiance model, which describes at-sensor observed radiance. The background emissivity and plume/ground temperatures are isolated, and their effects on chemical signal are described. This analysis shows that the plume's physical state, emission or absorption, is directly dependent on the background emissivity and plume/ground temperatures. It then describes what conditions on the background emissivity and plume/ground temperatures have inhibiting or amplifying effects on the chemical signal. These claims are illustrated by analyzing synthetic hyperspectral imaging data with the adaptive matched filter using two chemicals and three distinct background emissivities.

## 1. Introduction

Remotely detecting and identifying weak gaseous plumes using infrared measurement instruments is a challenge that receives continual attention. Burr and Hengartner [1] have provided a comprehensive review of this problem. Generally, the ability to detect a gaseous effluent is influenced by its concentration pathlength, atmospheric interferences, the temperature difference between the plume and the background surface, the emissivity of the background, and the complexity of the background surface. Collectively, these elements are termed clutter.

When trying to detect and identify gaseous effluents, the type and variability of background clutter in an image presents many modeling challenges. This subject is complex and has been studied from many perspectives. There have been several studies that address the characterization of background clutter [2, 3]. Endmember estimation is another method that models the types of clutter in an image on a subpixel (multiple background types within a pixel) level [4, 5]. There are also several studies that address signal-to-noise (SNR) and signal-to-clutter (SCR) ratios [6, 7]. These terms are used to represent the performance of least squares approaches to gas detection.

This paper studies the background clutter from a different perspective. We explore the effect of the background emissivity on the chemical signal as influenced by the temperature emissivity (*TE*) contrast; that is, we identify when the background emissivity will have inhibiting effects on the signal strength and in turn reduce SCR. We view the temperature emissivity contrast as a difference in radiance signal contribution of the plume and the background. First, we isolate the *TE* in a formulation of the physics-based radiance model. Then, we conduct analyses of the *TE's* contribution to the chemical signal while isolating the effect of the background emissivity. We describe the contributions of these terms to the chemical signal and identify cases where the plume/ground temperatures and background emissivity have amplifying/inhibiting contributions to the signal. To illustrate our claims, we simulate and analyze simplified hyperspectral images using the InfraRed Systems Analysis in General Environments (IR-SAGE) code [8–10].

This paper is organized as follows. Section 2 states the physics-based radiance model along with the analyses of the temperature emissivity contrast. Section 3 describes image simulation and gas detection methods. Section 4 presents results of the simulation studies. Section 5 presents conclusions.

## 2. Physics-Based Radiance Model and Analysis

We explore the three-layer physics-based radiance model to gain insight into how the structure of the background emissivity impacts the at-sensor observed plume signal [1, 11, 12]. This model can be written as

(1) $$L_{\text{obs}}(\nu )=\tau_{a}(\nu )\left[\left(1-\tau_{p}(\nu ))B(T_{p};\nu )+\tau_{p}(\nu )L_{g}(\nu \right)\right]+L_{u}(\nu )+e(\nu )$$

where *L_obs_* (*ν*) represents sensor-recorded radiance in *W*/*cm*^2^*Sr*cm^−1^ at wavenumber *ν* (*cm*^−1^),*τ_a_*(*ν*) and *τ_p_*(*ν*) are dimensionless terms representing the atmosphere and plume transmissivity, respectively, *B*(*T; ν*) has radiance units and is Planck's Blackbody function at wavenumber *ν* and temperature *T* (K), *L_g_*(*ν*) and *L_u_*(*ν*) are the ground-leaving and atmospheric upwelling radiances, respectively, and *e*(*ν*) includes unmodeled effects and sensor noise [2, 3].

Following the convention of [1, 13], we model the ground-leaving radiance as

(2) $$L_{g}(\nu )=\epsilon_{g}(\nu )B(T_{g};\nu )$$

where *ϵ_g_*(*ν*) is a dimensionless quantity representing the emissivity of the ground at wavenumber *ν*, and 0 ≤ *ϵ_g_*(*ν*) ≤ 1. Note that this formulation ignores the reflected atmospheric downwelling radiance. This assumption is reasonable in the Longwave Infrared band (LWIR) because the reflected radiance contribution to observed signal is negligible [13].

The Beer-Bourger-Lambert Law [14] gives an explicit expression for the transmissivity of a gas in terms of the chemical effluent's concentration path-length, *c* (with *c* measured in parts-per-million-meter, denote *dppm-m*), as follows:

(3) $$\tau_{p}(\nu )=e^{-A(\nu )c}$$

where *A*(*ν*) is the absorbance coefficient of the gas in *ppm-m*^−1^ [14]. For optically thin plumes, this term is well approximated by the first two terms in a Taylor Series expansion [1]. This gives

(4) $$\tau_{p}(\nu )\approx 1-A(\nu )c$$

for small *c*.

We now substitute Eq. (2) and Eq. (4) into Eq. (1) to arrive at the working-gas-plume linear model

(5) $$L_{\text{obs}}(\nu )=\tau_{a}(\nu )\left[B(T_{P};\nu )-\epsilon_{g}(\nu )B(T_{g};\nu )\right]A(\nu )c+\tau_{a}(\nu )\epsilon_{g}(\nu )B(T_{g},\nu )+L_{u}(\nu )+e(\nu )$$

which we interrogate further.

The right hand side of Eq. (5) shows that atmospheric radiance observed by the sensor is an additive layering of upwelling radiance *L_u_*(*ν*), ground radiance attenuated by atmosphere *τ_a_*(*ν*)*L_g_*(*ν*), and the signal due to the chemical plume *τ_a_*(*ν*) [*B*(*T_p_*; *ν*) − *ϵ_g_*(*ν*)*B*(*T_g_*; *ν*)]*A*(*ν*)*c*. This representation has been used to motivate scene whitening and the use of least squares approaches for gas detection [1, 12]. We will use this formulation to explore the effect that background emissivity and the ground/plume temperatures have on the chemical signal.

The radiance due to the chemical plume is the first term on the right hand side of Eq. (5), namely

(6) $$\tau_{a}(\nu )\left[B(T_{p};\nu )-\epsilon_{g}(\nu )B(T_{g};\nu )\right]A(\nu )c.$$

We will refer to Expression (6) as the *chemical signal* throughout the remainder of the paper. Inspection of this term shows that radiance due to the plume is a function of atmospheric transmission, plume and ground temperature, ground emissivity, and the plume gas absorbance and concentration path length. We note that while Expression (6) is one possible formulation for the chemical signal, there may be other approaches; for example, alternate formulations arise when log-transformations and linear mixture models are used. In this paper, we do not consider alternate modeling regimes. Thus, it is important to note that the conclusions we draw from the formulation given in Expression (6) are only pertinent to the linearization that we have applied in this analysis. We are interested in how the structure of the background emissivity affects the chemical signal. Toward that end, we will make the following assumptions:

1. The gas exhibits absorbance at wavenumber *ν*.
2. The gas is present at some fixed concentration path length *c*.
3. The atmospheric transmission, *τ_a_*(*ν*) is such that all plume radiance is not being attenuated by atmosphere at wavenumber *ν*.

Assumptions 1 and 2 confirm that there is a chemical signal (gas absorbance or concentration path length of 0 imply chemical signal of 0). Assumption 3 confirms that chemical radiance can pass through the atmosphere and reach the sensor.

To isolate the effects of background emissivity on the chemical signal at a single channel, we will focus our attention on *the Temperature Emissivity Contrast* that is defined below

(7) $$\text{TE}(T_{P},T_{g},\epsilon_{g},\nu )=B(T_{P};\nu )-\epsilon_{g}(\nu )B(T_{g};\nu ).$$

Expression (6) shows that the chemical signal is proportional to *TE*. Thus, larger values of *TE* (in absolute value) will yield larger chemical signals at wavenumber *ν*, making the plume easier to detect at that wavenumber. Therefore, we investigate the properties of the background emissivity *ϵ_g_*(*ν*) that will yield a larger *TE*. We consider three plume-ground temperature cases: *T_p_* = *T_g_*, *T_p_* > *T_g_*, and *T_p_* < *T_g_*. We analyze *TE* for each of these temperature cases in the next sections.

### 2.1. Case 1: T_p_ = T_g_

This case is presented first since it is the easiest to interpret analytically. Equality of the plume and ground temperatures implies that *B*(*T_p_*; *ν*) = *B(T_g_*; *ν*) = *B*(*T; ν*) for the common temperature *T*. Thus, when *T_p_* = *T_g_* = *T*, we can express Eq. (7) as

(8) $$\begin{array}{lll} \text{TE}(T_{p},T_{g},\epsilon_{g},\nu ) & = & B(T_{P};\nu )-\epsilon_{g}(\nu )B(T_{g};\nu )\\ & = & B(T;\nu )-\epsilon_{g}(\nu )B(T;\nu )\\ & = & \left(1-\epsilon_{g}(\nu ))B(T;\nu \right) \end{array}$$

Since 0 ≤ *ϵ_g_*(*ν*) ≤ 1, Eq. (8) demonstrates that a smaller *ϵ_g_*(*ν*) will result in a larger *TE* and hence a larger chemical signal. We also note that *TE* is strictly positive for this temperature case. An *ϵ_g_*(*ν*) = 1 is required for *TE* = 0, but this happens only for a true Blackbody. This indicates that the plume is in emission (emitting radiation) when *T_p_* = *T_g_*. A gas plume in emission is contributing to the observed radiance signal, and the chemical may be seen as peaks on the observed radiance at wavenumbers where the gas exhibits positive values in *A*(*ν*).

### 2.2. Case2: T_p_ > T_g_

When *T_p_* > *T_g_*, it follows that *B*(*T_p_*; *ν*) > *B*(*T_g_*; *ν*) since Planck's function is monotonically increasing with respect to *T*. We can write the plume's Blackbody radiance as a function of the ground's Blackbody radiance. We express this as

(9) $$B(T_{p};\nu )=1+\delta_{1}(\nu ))B(T_{g};\nu )$$

where we use *δ*_1_(*ν*) to represent the relative difference between the plume and ground Blackbody functions as follows:

(10) $$\delta_{1}(\nu )=\frac{B(T_{p};\nu )-B(T_{g};\nu )}{B(T_{g};\nu )}.$$

Substituting Eq. (9) into Eq. (7) yields

(11) $$\begin{array}{ll} \text{TE}(T_{p},T_{g},\epsilon_{g},\nu ) & =B(T_{P};\nu )-\epsilon_{g}(\nu )B(T_{g};\nu )\\ & =(1+\delta_{1}(\nu ))B(Tg;\nu )-\epsilon_{g}(\nu )B(Tg;\nu )\\ & =(1+\delta_{1}(\nu )-\epsilon_{g}(\nu ))B(Tg;\nu ) \end{array}$$

Eq. (11) is also easy to interpret as a function of *ϵ_g_*. First, we note that because *δ*_1_ (*ν*) is positive when *T_p_* > *T_g_* and is additive in *TE*, it can be shown that *TE* is strictly positive for this temperature case. This says that the plume is strictly in emission for this case. It also says that a larger chemical signal is observable compared to the previous temperature case. Since *ϵ_g_*(*ν*) is subtracted in Eq. (11), small emissivities yield a larger chemical signal at wavenumber *ν*.

### 2.3. Case3: T_p_ < T_g_

This temperature case is the most difficult to interpret analytically. When *T_p_* < *T_g_*, it follows that *B*(*T_p_*; *ν*) < *B*(*T_g_*; *ν*). Similar to the previous section, we can write

(12) $$B(T_{P};\nu )=(1-\delta_{2}(\nu ))B(T_{g};\nu )$$

where the *δ*_2_(*ν*) is now expressed as

(13) $$\delta_{2}(\nu )=\frac{B(T_{g};\nu )-B(T_{p};\nu )}{B(T_{g};\nu )}.$$

Note that the order of the Blackbody functions in Eq. (13) has changed from Eq. (10) to maintain a positive *δ*_2_(*ν*) function for this temperature case.

We substitute Eq. (12) into Eq. (7) and find that

(14) $$\begin{array}{ll} \text{TE}(T_{p},T_{g},\epsilon_{g},\nu ) & =B(T_{P};\nu )-\epsilon_{g}(\nu )B(T_{g};\nu )\\ & =(1-\delta_{2}(\nu ))B(Tg;\nu )-\epsilon_{g}(\nu )B(Tg;\nu )\\ & =(1-\delta_{2}(\nu )-\epsilon_{g}(\nu ))B(Tg;\nu ) \end{array}$$

The fact that *δ*_2_(*ν*) is a subtracted term in Eq. (14) complicates the interpretation of what properties of *ϵ_g_*(*ν*) are desirable for a larger *TE* and larger chemical signal. We provide a graphical summary of *TE* for the three temperature cases in the next subsection to clarify how *ϵ_g_*(*ν*) contributes to *TE* for each temperature case. For this temperature case, *TE* can be positive, negative, or 0, and this is directly dependent on *ϵ_g_*(*ν*) as well as *T_p_* and *T_g_*. A plume in absorption (*TE* < 0) is decreasing the observed radiance signal at wavenumbers where *A*(*ν*) is positive. In this case, the gas can be seen as troughs in the observed radiance. A plume that is neither emitting nor absorbing (*TE* = 0) is transparent to the sensor and cannot be detected.

### 2.4. Graphical Summary of TE for the three temperature cases

**Figure 1.**
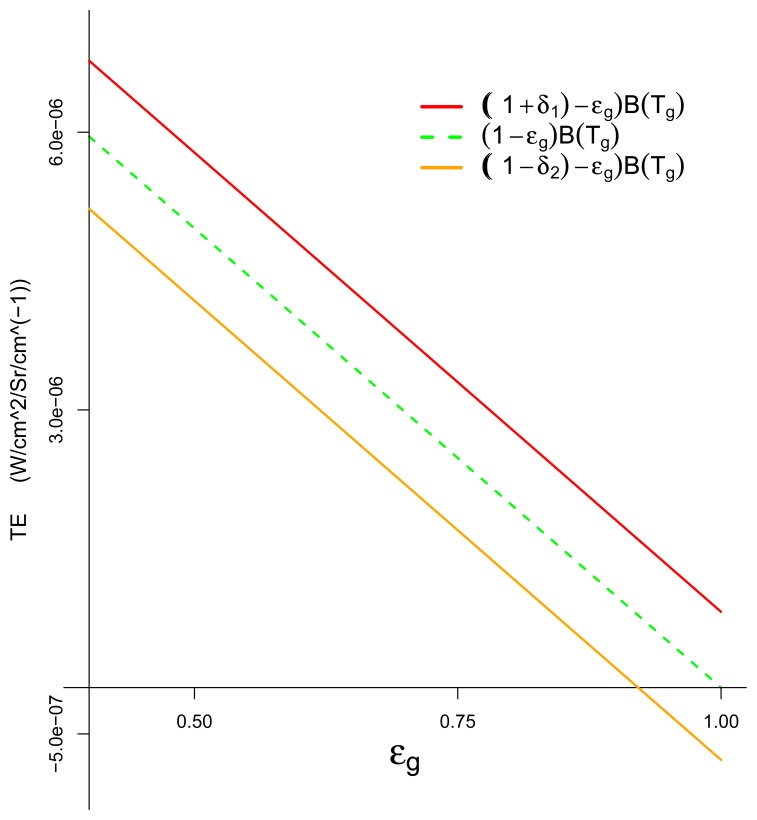
Temperature emissivity contrast plotted as a function of emissivity for three temperature cases at *ν* = 1000 cm^−1^: 305*K* = *T_p_* > *T_g_* = 300*K* (red line), *T_p_* = *T_g_* = 300*K* (green dashed line), and 295 = *T_p_* < *T_g_* = 300*K* (orange line). *TE* is non-negative when *T_p_* ≥ *T_g_*, which indicates the plume is in emission. *TE* can be positive or negative, depend-ing on *ϵ_g_* when *T_p_* < *T_g_*, which indicates the plume can be in emission or absorption.

We present a plot of *TE* as a function of *ϵ_g_*(*ν*) for the three temperature cases at *ν* = 1000 cm^−1^ in Figure 1. We set the ground temperature to *T_g_* = 300*K* and vary the plume temperature at *T_p_* = 305, 300, 295*K*.

The plots of *TE* for the *T_p_*> *T_g_* and *T_p_* = *T_g_* cases are the red and green lines, respectively. The plot shows that values of *ϵ_g_*(*ν*) closer to 0 increase the magnitude of *TE*. This is consistent with the interpretation of the analyses.

The plot of *TE* when *T_p_* < *T_g_* illustrates which values of *ϵ_g_*(*ν*) yield a larger *TE* in magnitude. We can see that *TE* crosses the horizontal axis if *ϵ_g_*(*ν*) = *B*(*T_p_*; *ν*)/*B*(*T_g_; ν*). This shows that when *ϵ_g_*(*ν*) = *B*(*T_p_; ν ν*/*B*(*T_g_; ν*), the plume is neither emitting nor absorbing and there is no chemical signal at wavenumber *ν*. The fact that *TE* can cross the horizontal axis informs us that small emissivities (closer to 0) *or* larger emissivities (closer to 1) have the potential to make the absolute value of *TE* larger for this temperature case.

The implications of this plot are as follows. When the gas exhibits an absorbance feature at wavenum-ber *ν* and *TE* is positive the plume is in emission. The analysis implies that backgrounds that have emissivities closer to 0 at wavenumber *ν* will then contribute to a larger chemical signal, and thus the plume will be easier to detect. We note that this is true for each temperature case *T_p_* = *T_g_* and *T_p_* > *T_g_*. When *T_p_* < *T_g_* it is possible for *TE* to be either positive or negative (plume is in emission *or* absorption). This is illustrated by the orange line in Figure 1. Thus, for this case, there are two ways that background emissivity can contribute to a larger *TE*. If *TE* is positive then emissivities closer to 0 yield a larger *TE* and a larger chemical signal. When *TE* is negative then emissivities closer to 1 may contribute to a larger chemical signal. Thus the plume will give some chemical signal for emissivities near 1 when *T_p_* < *T_g_* and *TE* < 0 and this distinguishes this temperature case from those where *T_p_* ≥ *T_g_*.

### 3. Experimental Methods

The goal of this section is to explore the validity of the analysis presented in Section 2. While the analysis describes the effect of *ϵ_g_*(*ν*) on the chemical signal at a single channel, hyperspectral instruments record a radiance vector across the LWIR band. As such, we will explore how these phenomena affect gas detectability in a multivariate setting. To illustrate the claims made in the single channel analysis, we will restrict ourselves to a specific subset of gases and background emissivities. We choose gases that exhibit strong absorbance over a small range of wavenumbers and no absorbance everywhere else. We also select emissivities that do not change ordering over the wavenumbers where the gas exhibited absorbance. The intent is that illustrating the phenomena with gases and backgrounds that exhibit this relationship will allow for immediate interpretation of the results in relation to the model analysis provided in section 2. We intend to explore the effects of general background emissivity and gas absorbance variability on gas detectability in future work.

**Figure 2.**
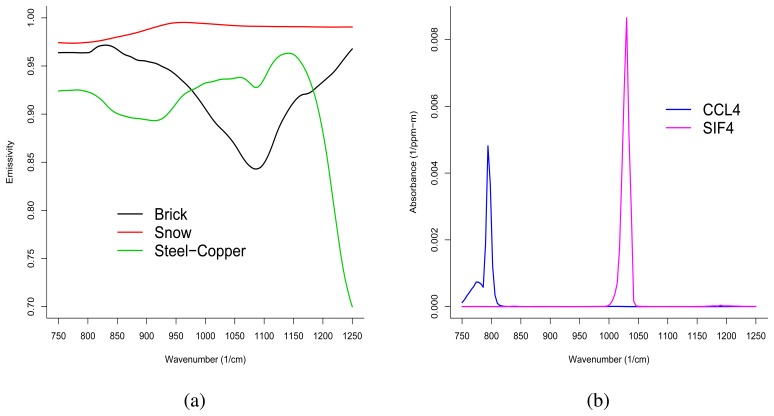
(a) Background emissivity spectra used in image simulation. (b) Chemical Ab-sorbance spectra used in image simulation.

We employ IR-SAGE to simulate simplified hyperspectral images. The background spectra used in this study are laboratory-measured individual background materials from the Nonconventional Exploita-tion Factors Data System (NEFDS), a government database of surface reflection parameters. We selected three distinct background emissivity clusters and used the mean spectra of the three clusters in image simulation. These spectra are representative of the following three groups: Brick, Snow, and Steel-Copper Tubing. These spectra are presented in Figure 2(a). Note that in the LWIR (750-1250 cm^–1^), these emissivities are relatively high.

Two gases were selected for image simulation. We selected Carbontetrachloride (CCL_4_) and Tetraflo-urosilane (SiF_4_). These gases exhibit one large dominant absorbance feature. A plot of the absorbance spectra for these gases is presented in Figure 2(b). The spectrum for CCL_4_ shows a major feature around 790 cm^–1^. SiF_4_ exhibits a dominant feature around 1025 cm^–1^. Note that the background emissivities show a consistent ordering across type (they do not cross) where each of the chemicals exhibits positive absorbance.

The simulated images have dimensions 75 × 120 × 126 (rows by columns by spectral dimension). The wavenumber range used is 750 to 1250 cm^–1^ in steps of 4. The three background spectra are inserted across the rows in three 25-pixel swaths. The chemicals are inserted as six 20-column bands at concentration pathlengths 16, 8, 4, 2, 1, and 0 *ppm-m*. This orientation produces 500 pixel replicates within a background/gas concentration combination.

Images were created for each temperature case. The ground temperature, *T_g_*, was kept constant at 300K, and the plume temperature, *T_p_*, was varied at *T_p_* = 305*K*, 300*K*, and 295*K* for the CCL_4_ images. This temperature range was selected to illustrate the effect for each of the background emissivities on detection. A slightly wider temperature range was needed to illustrate the effect for SiF_4_: *T_g_* = 300 and *T_p_* = 305*K*, 300*K*, 292*K*.

Simulated sensor noise was used to perturb the spectra in each pixel. Variability due to atmosphere, temperature, and emissivity from pixel to pixel were held constant to enable us to study the effect of the background emissivity's variability across the spectrum on the chemical signal. IR-SAGE models sensor noise from a zero-mean Gaussian distribution with standard deviation defined by the ratio of sensor incident radiance to the signal-to-noise ratio. Please see [8–10] for detailed descriptions of the sensor noise model.

We use the Adaptive Matched Filter (*AMF*) as a gas detector, note that this is equivalent to the gener-alized least squares solution to a linear model e.g. see [1, 15]. The image analysis process is as follows. The non-gas pixels can be formulated as

(15) $$L_{\text{offi}}=\tau_{a}⊙L_{g}+L_{u}+e_{i},\;i=1,\ldots ,500$$

where bold terms are vectors of length 126 (126 spectral channels) and ⨀ denotes the Hadamard product (elementwise multiplication;. We compute the mean of these pixels for use in background radiance subtraction. This can be represented as $\bar{L_{\text{ofi}}}=\tau_{a}⊙L_{g}+L_{u}+\bar{e}$ for the 500 non-gas replicates as the atmospheric transmissivity and background radiance are not varied across pixels. We subtract this quantity from each of the gas pixels that contain the same background type, i.e., we compute

(16) $$L_{\text{obs}}-\bar{L_{\text{off}}}=\tau_{a}⊙\left[B(T_{p})-\epsilon_{g}⊙B(T_{g})\right]⊙Ac+e-\bar{e}.$$

Eq. (16) shows how the background mean subtraction removes radiance due to ground as well as atmo-spheric upwelling radiance and leaves the chemical signal and noise. These data are processed with the *AMF*. Explicitly we compute

(17) $$\text{AMF}={\left(A^{\prime }{\hat{\Sigma }}^{-1}A\right)}^{-1}A^{\prime }{\hat{\Sigma }}^{-1}(L_{\text{obs}}-\bar{L_{\text{off}}})$$

where *A* is a 126 × 1 vector of the gas absorbance spectra and ∑ˆ : 126 × 126 represents the spectral covariance matrix computed on the non-gas pixels. This formulation of the filter is sometimes used in practice in the LWIR. It assumes no information about the atmosphere, plume or ground temperatures, and emissivity is available. It also assumes that *TE* is constant across the spectral dimension [1, 2].

If the *AMF* is deemed statistically significant based on a 5% level two-sided hypothesis test [16], then we say we have “detected” the gas in that pixel. This test is conducted under the assumption that the errors follow a Gaussian distribution. We apply this solution to each of the 500 replicates within each gas concentration path-length/background combination and then record the proportion of detections:

(18) $$\hat{p}=\frac{\#\text{of detections}}{500}.$$

We use *pˆ* as an estimate of the gas detection probability. We get 18 detection probabilities for each image as there are three backgrounds and six gas-concentration path-length levels. Lastly, we plot detection proportions versus concentration path length and look for orderings (conditioned on background emis sivity) in these curves. We then interpret these results back to the claims to the effect that background emissivities should produce a larger chemical signal and better detectability for each chemical.

### 4. Results

**Figure 3.**
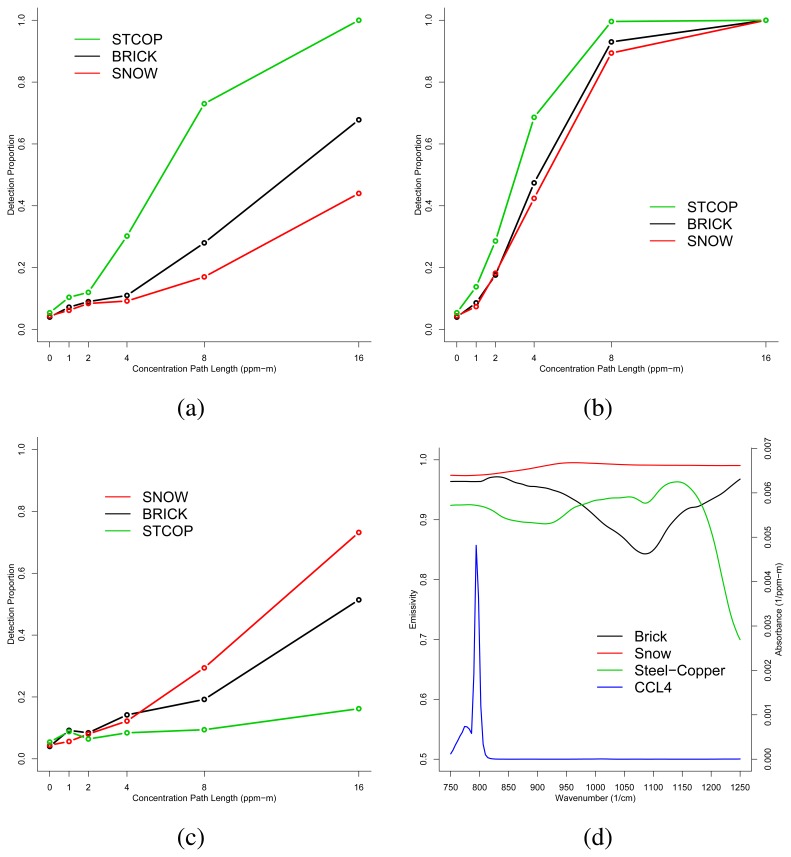
Detection proportions for CCL_4_ when (a) *T_p_* = *T_g_* = 300*K*, (b) *T_p_* = 305*K* > *T_g_* = 300*K*, (c) *T_p_* = 295*K* < *T_g_* = 300*K*, and (d) gas absorbance spectra and background emissivities.

**Figure 4.**
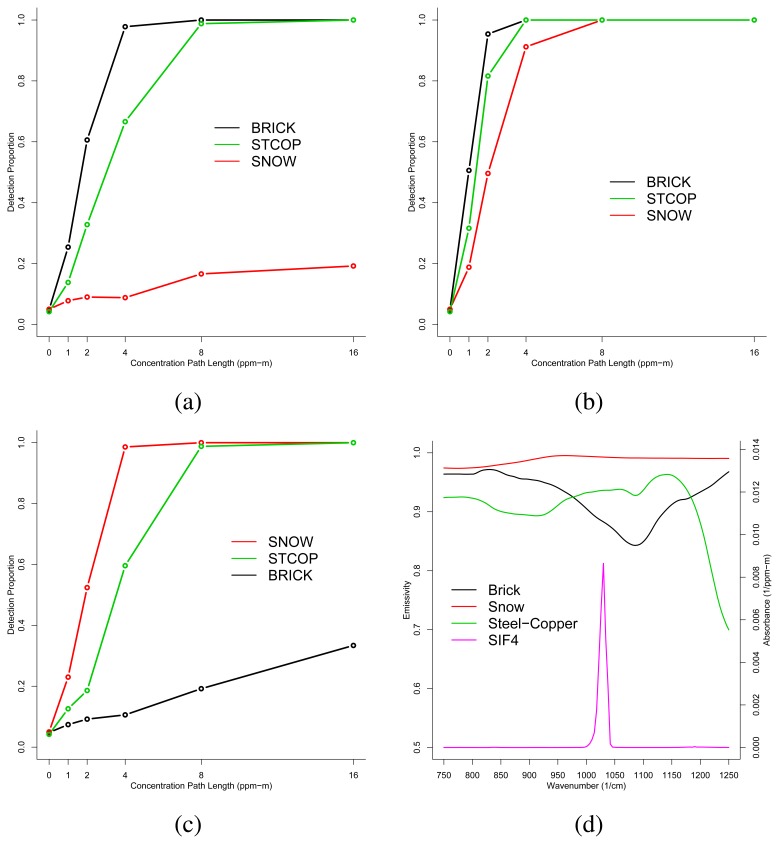
Detection proportions for SiF_4_ when (a) *T_p_* = *T_g_* = 300*K*, (b) *T_p_* = 305*K* > *T_g_* = 300*K*, (c) *T_p_* = 292*K* < *T_g_* = 300*K*, and (d) gas absorbance spectra and background emissivities.

In this section, we present the results of analyses performed on the six synthetic images that were generated using IR-SAGE. Sample detection proportions were computed for each combination of con-centration path-length and background type. Taken together, these data form the empirical detection proportion curves presented in Figure 3 (for CCl_4_) and Figure 4 (for SiF_4_).

First, we consider Figure 3(a), which is a plot of the empirical detection curves for CCL_4_ when *T_p_* = *T_g_* = 300*K*. Examination of the plot shows that the backgrounds can be ordered by detection proportion as (best to worst) Steel-Copper, Brick, and Snow. Figure 3(d) shows a plot of the CCL_4_ absorbance spectra along with the background emissivities. We see that where CCL_4_ exhibits the large absorbance peak, the background emissivities can be ordered from low to high as Steel-Copper, Brick, and Snow. Thus, the results here are consistent with the characterization of *TE* for this temperature case: lower background emissivities contribute to larger chemical signal when the plume is in emission.

Next, we consider the detection proportion plot for 305*K* = *T_p_* > *T_g_* = 300*K* in Figure 3(b) which shows the same ordering in backgrounds as the previous temperature case. It also shows that detection has generally increased over all backgrounds. These observations are consistent with the characterization of *TE* as they represent the fact that the backgrounds that give better detection for the *T_p_* = *T_g_* case are the same as for the *T_p_* > *T_g_* case. They also represent the fact that *T_p_* > *T_g_* gives a larger *TE* and in turn a larger chemical signal.

Third, we consider Figure 3(c), which shows the empirical detection curves for CCL_4_ when 295*K* = *T_p_* < *T_g_* = 300*K*. Now we see that the ordering in the detection curves by background is Snow, Brick, and then Steel Copper. Inspection of Figure 3(d) shows that the background emissivites can be ordered (high to low) as Snow, Brick, then Steel-Copper where CCL_4_ exhibits its dominant absorbance peak. These results are consistent with the analysis of *TE* for this temperature case. They illustrate that, for an absorbing plume and a large enough (negative) *TE*, the backgrounds that give best detection are those that exhibit larger emissivities and not smaller ones as in the previous two temperature cases.

Now we consider Figure 4(a), which is a plot of the empirical detection curves for SiF_4_ when *T_p_* = *T_g_* = 300*K*. Examination of the plot shows that the backgrounds can be ordered from best to worst detectability for SiF_4_ as Brick, Steel-Copper, and Snow. Figure 4(d) shows that the background emissivities can be ordered from least to greatest as Brick, Steel-Copper, and Snow where SiF_4_ exhibits its dominant absorbance peak. This ordering is consistent with the analysis of *TE*: smaller emissivities yield a larger chemical signal for this case.

Next, we consider Figure 4(b), which gives the SiF_4_ detection proportions when 305*K* = *T_p_* > *T_g_* = 300*K*. Examination of this plot shows that the background orderings by detection proportion are the same as the *T_p_* = *T_g_* case. The plot also shows that detection has generally increased for the emitting plume at a higher temperature.

Last, we consider the plot in Figure 4(c), which presents the empirical detection proportions when *T_p_*= 292*K* < *T_g_* = 300*K*. This plot shows that the backgrounds that yield best detection have changed ordering to Snow, Steel-Copper, and Brick. Again, we see that larger emissivities yield larger detection proportions for a large *TE* when *T_p_* < *T_g_*.

### 5. Conclusions

The effects of clutter on gas plume detection/identification is a complicated problem that is ap-proached from multiple perspectives. This paper studied the effects of background emissivity and plume/ground temperatures on the chemical signal. The analysis is most pertinent to treatments of the physics-based radiance model that linearize the plume transmissivity term and work with linear ap-proaches to gas detection or identification.

Our investigation has shown that, when ignoring reflected downwelling radiance, the physical state of the plume (emission, neutral, or absorption) is not only dependent on the plume/ground temperatures, but is also directly dependent on the background emissivity, ϵ*_g_* (*ν*), at a particular wavenumber. We have shown that *T_p_* ≥ *T_g_* implies that the plume is strictly in emission and that values of ϵ*_g_* (*ν*) closer to 0 will contribute to a larger observed chemical signal. Further, when *T_p_* < *T_g_*, we have shown that it is possible that the plume is in emission, absorption, or neither emitting nor absorbing (neutral). A neutral plume happens when ϵ*_g_* (*ν*) = *B*(*T_p_*; *ν*)/*B*(*T_g_*; *ν*). Thus, the background has the potential to completely obscure the plume at wavenumber *ν*. The analysis also shows that emissivities closer to 0 or 1 in this case have the potential to contribute to a larger observed chemical signal.

The analysis was verified by analyzing simulated hyperspectral radiances in the absence of atmo-spheric, background, and temperature variability. The gas absorbance and background emissvities used in image simulations exhibited a very strong relationship: the background emissivity ordering did not change over the wavenumbers that exhibited gas absorbance. This made it possible to illustrate the re-sults of the model analysis in a multivariate setting. In general, real background emissvities and gas absorbances will not exhibit this relationship, so we aim to further explore this phenomenology while allowing the gas absorbance and background emissivity to vary across the entire LWIR portion of the spectrum.
